# Assessing resilience in adolescence: The Spanish adaptation of the Adolescent Resilience Questionnaire

**DOI:** 10.1186/s12955-015-0259-8

**Published:** 2015-07-11

**Authors:** Georgina Guilera, Noemí Pereda, Ana Paños, Judit Abad

**Affiliations:** Department of Behavioural Sciences Methodology, Faculty of Psychology, University of Barcelona, Passeig Vall d’Hebron 171, 08035 Barcelona, Spain; Research Group on Child and Adolescent Victimization (GReVIA), University of Barcelona, Barcelona, Spain; Institute for Brain, Cognition and Behaviour, University of Barcelona, Barcelona, Spain; Department of Personality, Assessment and Psychological Treatment, Faculty of Psychology, University of Barcelona, Passeig Vall d’Hebron 171, 08035 Barcelona, Spain; Specialisterne, S.L., Esadecreapolis, Avinguda de la Torre Blanca 57, 08172 Sant Cugat del Vallès, Barcelona, Spain

**Keywords:** Resilience, Adolescence, Assessment, Adaptation, Spain

## Abstract

**Background:**

The concept and assessment of resilience have attracted considerable attention in recent years, but none of the instruments developed to measure resilience in adolescents have been adapted to the Spanish context. The Adolescent Resilience Questionnaire (ARQ) provides a comprehensive and multidimensional assessment of the resources associated with resilience in adolescents.

**Methods:**

This study analyzes the psychometric properties of the ARQ. Participants included a community sample of 1101 Spanish adolescents (53.5 % boys) aged 12–17 years (*M* = 14.51; *SD* = 1.755).

**Results:**

Results confirm the factor structure based on 12 scales. Internal consistency was generally adequate (between .60 and .84), although the unacceptable coefficient for the *Empathy/Tolerance* scale (α = .38) means that this scale needs to be revised for the Spanish context. Relationships between ARQ scales and psychopathology were in the expected direction and magnitude. Some gender differences were observed, with higher scores for boys on *Confidence* and *Negative cognition*.

**Conclusions:**

The Spanish version of the ARQ can help to identify personal characteristics associated with resilience and signs of positive engagement with family, peers, school, and the community. It can identify those adolescents most likely to show resilience in response to adversity, as well as those who may be vulnerable under situations of stress.

**Electronic supplementary material:**

The online version of this article (doi:10.1186/s12955-015-0259-8) contains supplementary material, which is available to authorized users.

## Resumen

**Antecedentes:**

El concepto de resiliencia y su evaluación han cobrado especial interés en los últimos años, si bien existen escasos instrumentos que midan la resiliencia en adolescentes y ninguno adaptado al contexto español. El Adolescent Resilience Questionnaire (ARQ) evalúa recursos asociados a la resiliencia en población adolescente desde una perspectiva comprehensiva y multidimensional.

**Métodos:**

Este estudio analiza las propiedades psicométricas del ARQ. Los participantes fueron una muestra comunitaria de 1,101 adolescentes españoles (53.5 % chicos), de entre 12 y 17 años (*M* = 14.51; *DT* = 1.755).

**Resultados:**

Los resultados confirman la estructura factorial formada por 12 escalas. Los valores de consistencia interna fueron en conjunto adecuados (entre .60 y .84), aunque la escala *Empathy/Tolerance* (α = .38) presenta un coeficiente de consistencia inaceptable y debería revisarse para el contexto español. Las relaciones entre las escalas del ARQ y la psicopatología fueron en la dirección y magnitud esperadas. Se observan diferencias de género en la capacidad de resiliencia, con mayores puntuaciones en los chicos para las escalas *Confidence* y *Negative cognition*.

**Conclusiones:**

La versión española del ARQ puede ser útil para determinar características personales asociadas a la resiliencia y vínculos positivos con la familia, amigos, escuela y comunidad, permitiendo identificar adolescentes más proclives a mostrar resultados resilientes en situaciones de adversidad, así como aquellos más vulnerables ante situaciones de estrés.

## Background

Understanding resilience is important for society and it continues to be the object of study in relation to its potential influence on health, well-being, and quality of life [1]. Despite the increasing number of scientific papers that have addressed this construct in recent decades [2], several controversies and questions remain as regards its conceptualization, and this has direct implications for its measurement. These problems include the lack of consensus over its definition [3, 4], the possibility of considering it either as a positive outcome in the face of adversity or as a dynamic process of adaptation to risk [5], its relationship to the presence of specific risk and protective factors [6], resilience as global or multidimensional [7], and the question of whether it should be regarded as a stable or dynamic construct [8–10].

These historical controversies make assessment of the construct more difficult, as all the variables that contribute to its definition should ideally be included in a single instrument that is capable of providing a comprehensive and exhaustive measure. In fact, however, the range of different perspectives has led to a multiplicity of instruments, thus hampering consensus and the comparison of results. Although improvements to these instruments have been made over time, a number of methodological limitations remain [11]. In this regard, one of the most recent systematic reviews of measures of resilience [12] highlights that most of the instruments developed to date generally focus on the individual level, rather than considering all the contexts that have been shown to be relevant to resilience, such as the family, the community environment, or social relations. In addition, the instruments with the best psychometric properties are targeted at adults, the population for which most such measures have been designed.

The assessment of resilience in adolescents is, by comparison, a neglected area of study (see the review of instruments for this population by Ahern and colleagues [13]), although it is generally agreed that resilience is related to vulnerability and resistance to psychopathology in this age group [14, 15]. Furthermore, measures of resilience have been linked to internalizing problems as anxiety and depression [16], with slightly higher correlations between these variables being observed among girls than boys [17]. Resilience has been usually understood as the absence of psychopathology or the demonstration of competence within an adverse environment [18, 19]. However, it is necessary to consider that the protective character of a resilience factor in one specific domain is not a guarantee for the adolescent adjustment in other competence spheres and for other psychopathological problems [20]. Nonetheless, few studies have sought to develop instruments specifically for adolescents, despite the obvious interest in understanding how young people respond to adversity [5] and, therefore, the importance of assessing resilience in this age group [21].

Consequently, there is a need for instruments that are able to provide an accurate assessment of this construct from a broad perspective while also taking into account the specific features of this population. One of the most comprehensive measures for assessing resilience in adolescents is the *Adolescent Resilience Questionnaire* (ARQ) [22], which was developed in an attempt to provide a psychometrically robust instrument that encompasses not only individual characteristics but also all those variables that have been shown by research to contribute to resilience. The ARQ is based on an ecological-transactional model that regards individual and environmental factors as having a mutual influence on each other [23], a notion that is consistent with the most recent definitions of resilience [4]. This integration of the personal and environmental components of resilience allows a broader examination of the construct as a complex and multidimensional phenomenon, thus favoring the development of more effective intervention programs [24]. Within this model, each domain, such as the cultural context, the neighborhood or school setting, or the family contains risk and protective factors (lasting or transitory) for the individual. Consequently, resilience implies an interaction between risk variables, protective factors, and the intervention that is offered to the adolescent, which must be based on a correct assessment of his or her resources [21].

### Goals and hypothesis

Given the importance of culture in any assessment of resilience [25] the aim of the present study is to conduct the first adaptation of an instrument that has been shown in the English-speaking world to be one of the best available measures of adolescent resilience (i.e., in terms of being developmentally appropriate and covering multiple domains), and to analyze its suitability for the cultural context of a southern European country. In addition to the dimensional structure and internal consistency analyses, two other pieces of evidence concerning validity are assessed: a) relationship with psychopathology: even though studies have not been conclusive regarding the relationship between different types of resilience and the development of internalizing and externalizing symptoms, resilience and psychopathology have been shown to influence each other [14, 15], so we hypothesized that adolescents with higher global resilience scores will present lower levels of symptoms of psychopathology, both internalizing and externalizing [26]; and b) differences between genders: since previous studies have reported significant sex differences when assessing resilience in adolescents [17], we also examine whether boys and girls differ on this variable.

## Methods

### Participants

Participants were children and adolescents aged between 12 and 17 years old recruited from seven high schools in a region of north-east Spain, covering different socioeconomic levels (low, medium, and high). A convenience sampling was used for choosing schools, and in order to cover all target ages classes within schools were randomly selected. Students with cognitive and/or language difficulties (less than 1 %), conditions which might undermine the validity of their responses to the assessment protocols, were excluded. The initial sample comprised 1,152 adolescents, of whom 51 (4.43 %) were eliminated due either to being older than 17 years of age or because at least 10 % of data were missing from their ARQ.

### Measures

*Adolescent Resilience Questionnaire*. The ARQ [22] is an 88-item self-administered screening instrument for adolescents aged between 11 and 19 years, and it assesses both individual and environmental factors underlying resilience. Items are responded to using a five-point Likert scale anchored by *Almost never* (1) and *Almost always* (5). The questionnaire includes 12 scales that measure resilience factors across five different domains (see Table 1).

**Table 1:** Structure and composition of the ARQ

Domain/Scale	Definition	Number of items	Example of item
1. Self			
Confidence	Self-confidence and future expectations	8	I feel confident that I can handle whatever comes my way
Emotional insight	Understanding and regulation of emotions	8	I think things through carefully before making decisions
Negative cognition (reversed scale)	Tendency to worry, to ruminate and to pessimism.	8	I tend to think the worst is going to happen
Social skills	Communication skills and ability to develop connections with others	8	I can express my opinions when I am in a group
Empathy/Tolerance	Capacity to understand others’ feelings and perspective	8	I am patient with people who can’t do things as well as I can
2. Family			
Connectedness	Nurturing and supportive family environment	8	I enjoy spending time with my family
Availability	Family members’ availability to offer support and advice	3	There is someone in my family I can talk to about anything
3. Peers			
Connectedness	Connections to friends and confidence with peers	7	I have friends I can trust with my private thoughts and feelings
Availability	Ability to develop and maintain friendships with peers	8	I wish I had more friends I felt close to
4. School			
Supportive environment	Support from teachers and school staff	8	My teachers are caring and supportive of me
Connectedness	Engagement with school socially and academically	8	I try hard in school
5. Community			
Connectedness	Perceptions of belonging to the community and support from people in their neighborhood	6	I trust the people in my neighborhood

The psychometric properties of the original ARQ were examined in a sample of 451 students from 11 secondary schools in Australia [22], revealing a 12-scale structure with adequate to good internal consistency coefficients (range between .70 and .90).

With the authors’ permission, the original items of the ARQ were translated into Spanish and Catalan by two child and adolescent psychologist experts and reviewed in terms of their comprehensibility by five native speakers and some adaptations and modifications were carried out. The back-translation procedure was applied, and both the original and the back-translated English versions were compared in terms of their psychological meaning. When discrepancies emerged they were discussed by the two experts, and the translated version was accordingly modified.

*Youth Self Report.* The Youth Self Report (YSR) [27] is designed to screen for psychopathology in adolescents aged between 11 and 18 years. The present study used only the second part of the questionnaire, which comprises 119 items each with three response options (0: *Not true*, 1; *Somewhat or sometimes true*; 2: *Very true or often true*). This problems scale covers both *internalizing* and *externalizing* problems, as well as what are referred to as *mixed* syndromes. Alpha values for the internalizing, externalizing, and total problems scales range between 0.85 and 0.90 [27]. Transcultural research has confirmed the dimensional structure of the YSR in a highly diverse range of countries [28].

### Procedure

Approval to carry out the study was sought from the Institutional Review Board of the University of Barcelona (IRB00003099). School principals and the parents or legal guardians of the adolescents were informed about the study’s objectives and their consent to participate was obtained. The adolescents themselves were also informed about the nature of the research, and they gave verbal consent being made clear that participation was voluntary and that all data would remain confidential at all times. Less than 3 % of the sample declined to take part of the study because parents refused consent. The instruments were completed during normal classroom time (i.e., 50 min) in the presence of two researchers. After completing the questionnaires participants were given the opportunity of a debriefing session in which they could ask questions and/or receive guidance.

### Data analysis

A confirmatory factor analysis was performed to explore the fit of the data to the dimensional structure proposed by the authors of the ARQ, namely 12 correlated scales (see Table 1).

The internal consistency of the instrument was evaluated by obtaining the Cronbach’s alpha coefficient for each scale. In order to explore the contribution of items to the internal consistency we also calculated item-corrected total score correlations for each scale, as well as the alpha coefficient were a given item to be eliminated. The relationship between resilience scores on the ARQ and psychopathology scores on the YSR was studied by computing the Pearson correlation coefficient for both the total sample and for boys and girls separately. Sex differences in resilience were examined by comparing the mean scores of boys and girls on the ARQ scales using multivariate analysis of variance, taking age and the level of general psychopathology as covariables. Statistical significance was set at *p* < .05, and the value of *η*^*2*^ was calculated as a measure of effect size.

Analyses were performed using SPSS v15, or in the case of the confirmatory factor analysis, AMOS v18.

## Results

### Sample description

The final sample consisted of 1101 adolescents (53.5 % boys and 46.5 % girls) aged 12–17 years (*M* = 14.51; *SD* = 1.76), the large majority of whom were Spanish nationals (94.7 % Spain; 3.6 % Central and South America; 0.8 % rest of Europe; 0.7 % Asia; 0.2 % Africa).

### Descriptive data and standardization

Table 2 presents general descriptive data for each of the ARQ scales, along with various percentiles as a way of standardizing scores. We also calculated the percentage of participants achieving the minimum and maximum score, in order to detect possible floor and ceiling effects, respectively. No such effects were observed, with the exception of the *Availability*-*Family* scale, where a considerable proportion of respondents (26.5 %) achieved the maximum score. This may indicate that this scale does not discriminate adequately between people with high levels on this aspect of resilience.

**Table 2:** Descriptive data and percentiles for the scales of the ARQ

Domain/Scale	Mean	SD	Theoretical min.-max. score	% Min.	% Max.	P_5_	P_10_	P_25_	P_50_	P_75_	P_90_	P_95_
1. Self												
Confidence	31.21	5.19	8-40	0.1	3.2	22	24	28	32	35	38	39
Emotional insight	28.50	4.51	8-40	0	0.1	21	23	26	29	32	34	35
Negative cognition	24.05	5.62	8-40	0.1	0.1	15	17	20	24	28	31	34
Social skills	27.26	5.40	8-40	0	0.6	18	20	24	27	31	34	36
Empathy/Tolerance	27.63	3.76	8-40	0	0	21	23	25	28	30	32	33
2. Family												
Connectedness	29.75	6.29	8-40	0.2	1.4	18	21	26	31	35	37	38
Availability	11.70	3.24	3-15	2.2	26.5	5	7	10	12	15	15	15
3. Peers												
Connectedness	30.21	4.16	7-35	0	13.7	22	25	28	31	33	35	35
Availability	31.38	5.18	8-40	0	1.7	21	24	28	32	35	37	39
4. School												
Supportive environment	26.49	6.07	8-40	0	0.7	16	19	22	27	31	34	36
Connectedness	27.58	5.17	8-40	0.1	0.3	18	21	24	28	31	34	35
5. Community												
Connectedness	19.92	5.32	6-30	1.1	3.0	10	13	16	20	24	27	29

### Dimensional structure

Given that the data did not fit the multivariate normal distribution (kurtosis value of one item above 7.00 and the *z* statistic of multivariate normality above 5.00 [29]) we used the unweighted least squares procedure, an adequate technique when there is a considerable number of items and sample size is high (e.g., [30]). Results of the confirmatory factor analysis (see Additional file 1) show that the proposed model of 12 scales presents an adequate fit, since the goodness-of-fit index (GFI) was .91 and the value of the standardized root mean square residual (SRMR) was .06 [31]. Factor loadings (lambda coefficients) for most of the items (87.5 %) were above .30, with most of those that failed to reach this threshold corresponding to the *Empathy/Tolerance* scale. Upon examining the distribution of residuals we observed that they followed a symmetrical distribution, with values below 0.10 in almost 90 % of cases.

Generally, the magnitude of the correlations between scales from the same domain was higher than that between scales from different domains (see Table 3). Noteworthy correlations for the same domain were those between *Confidence* and *Emotional insight* (*r* = .75) and between *Connectedness-Peers* and *Availability-Peers* (*r* = .80).

**Table 3:** Inter-correlations among ARQ scales

Domain/Scale		1. Self					2. Family		3. Peers		4. School	
		Co	EI	NC	SS	E/T	C	A	C	A	SE	C
1. Self	Co											
	EI	***.75***										
	NC	***.56***	*.29*									
	SS	***.61***	***.56***	***.51***								
	E/T	***.69***	***.62***	***.69***	***.59***							
2. Family	C	**.50**	**.56**	.27	.28	.50						
	A	.33	.49	.10	.25	.37	***.67***					
3. Peers	C	.47	**.62**	.16	**.62**	**.58**	.30	.32				
	A	.47	.39	.39	**.78**	**.61**	.24	.16	***.80***			
4. School	SE	.42	**.52**	.14	.36	.38	.41	.38	.38	.28		
	C	.42	.47	.14	.39	.33	.37	.23	.32	.24	***.70***	
5. Community	C	.33	.39	.16	.28	.37	.36	.29	.40	.33	.32	.18

Note: *Co* Confidence, *EI* Emotional insight, *NC* Negative cognition, *SS* Social skills, *E/T* Empathy/Tolerance, *C* Connectedness, *A* Availability, *SE* Supportive environment

Note: Correlations ≥ .50 are in bold. Correlations among scales from the same domain are in italics

### Internal consistency

Based on the criteria of Kline (2005), scores for the scales *Confidence* (α = .82), *Connectedness-Family* (α = .84), *Availability-Family* (α = .80), and *Connectedness-Community* (α = .82) present good internal consistency. The remaining scales show acceptable reliability indices, ranging between .70 and .78, the exception being the scales *Emotional insight* (α = .60) and *Empathy/Tolerance* (α = .38), whose internal consistency coefficients can be regarded as questionable and unacceptable, respectively (see Additional file 1). The item-corrected total score correlations (i.e., eliminating a given item score from the total score for the scale) were obtained and 79.54 % of the items presented values ≥ .30. However, none of the eight items on the *Empathy/Tolerance* scale fulfilled this criterion (see Additional file 1). It should be noted that for all the scales, eliminating the item with the lowest item-corrected total score correlation did not produce a considerable increase in the value of the Cronbach’s alpha coefficient.

### Relationship with psychopathology

Table 4 shows the Pearson correlation coefficients between the ARQ scales and the subscales of the YSR. These coefficients were evaluated according to the criteria proposed by Cohen [32].

**Table 4:** Correlations between ARQ scales and scores on the YSR

Domain	Scale	Internalizing problems			Externalizing problems			Total YSR		
		Boys	Girls	Total	Boys	Girls	Total	Boys	Girls	Total
1. Self	Co	*-.49*	**-.55**	**-.54**	-.26	*-.30*	-.27	*-.49*	**-.51**	**-.51**
	EI	-.23	*-.36*	-.29	-.20	-.23	-.21	-.23	*-.35*	-.29
	NC	**-.55**	**-.59**	**-.59**	-.24	*-.39*	*-.30*	*-.46*	**-.58**	**-.53**
	SS	*-.47*	**-.55**	**-.51**	-.08	-.08	-.08	*-.31*	*-.36*	*-.34*
	E/T	-.29	*-.41*	*-.32*	*-.37*	*-.37*	*-.37*	*-.36*	*-.46*	*-.39*
2. Family	C	-.20	*-.37*	-.29	*-.31*	*-.36*	*-.38*	*-.32*	**-.51**	*-.42*
	A	-.14	-.22	-.16	-.18	-.21	-.20	-.19	-.25	-.20
3. Peers	C	-.29	*-.41*	-.29	-.01	-.11	-.05	-.19	*-.30*	-.21
	A	*-.47*	*-.47*	*-.44*	-.08	-.09	-.09	-.*33*	*-.32*	*-.31*
4. School	SE	-.16	-.19	-.15	-.18	-.27	-.23	-.17	-.27	-.21
	C	-.23	-.24	-.18	*-.34*	*-.38*	*-.36*	*-.35*	*-.38*	*-.33*
5. Community	C	-.11	-.21	-.17	-.11	-.09	-.09	-.17	-.16	-.16

*Co* Confidence, *EI* Emotional insight, *NC* Negative cognition, *SS* Social skills, *E/T* Empathy/Tolerance, *C* Connectedness, *A* Availability, *SE* Supportive environment

In bold, high correlations, and in italics, moderate correlations, both according to the criteria of Cohen^28^

Internalizing problems showed a notable relationship with the scales *Confidence* (*r* = −.54), *Negative cognition* (*r* = −.59), and *Social skills* (*r* = −.51), all of which form part of the *Self* domain. Somewhat weaker associations were observed with *Empathy/Tolerance* (*r* = −.32) and *Availability-Peers* (*r* = −.44). None of the ARQ scales showed a strong relationship with externalizing problems, although there were moderate correlations with *Negative cognition* (*r* = −.30), *Empathy/Tolerance* (*r* = −.37), *Connectedness-Family* (*r* = −.38), and *Connectedness-School* (*r* = −.36). Finally, the total problems score on the YSR presented a similar pattern of correlations to those already mentioned, with notable relationships again being found with *Confidence* and *Negative cognition* (*r* = −.51 and *r* = −.53, respectively). *Availability-Family*, *Supportive Environment-School*, and *Connectedness-Community* showed low correlations with all three psychopathology scales.

It should be noted that, in general, the relationship between the resilience scales and the measures of psychopathology was stronger among girls than boys, especially with regard to internalizing and total problems.

### Differences between boys and girls

In terms of the level of resilience the analysis revealed a significant difference between the ARQ scores of boys and girls (*F*(12,599) = 15.93, *p* < .05, *η*^*2*^ = .24). Specifically, boys scored higher than girls on the scales *Confidence* (*F*(1,610) = 36.47, *p* < .05, *η*^*2*^ = .06), *Negative cognition* (*F*(1,610) = 41.97, *p* < .05, *η*^*2*^ = .06), and *Connectedness-Family* (*F*(1,610) = 6.14, *p* < .05, *η*^*2*^ = .01), although the magnitude of these differences was only notable for the first two of these scales, where the effect size was moderate [32]. Conversely, girls scored higher than boys on *Availability-Family* (*F*(1,610) = 6.26, *p* < .05, *η*^*2*^ = .01), *Connectedness-Peers* (*F*(1,610) = 14.30, *p* < .05, *η*^*2*^ = .02), and *Connectedness-School* (*F*(1,610) = 28.16, *p* < .05, *η*^*2*^ = .04), although none of these differences had a notable effect size [32].

## Discussion

This study shows that the ARQ is, generally speaking, an adequate instrument for assessing different aspects of resilience in Spanish adolescents. The fit of the data to the 12-scale model was found to be acceptable, thus confirming the dimensional structure proposed by the scale’s authors. The internal consistency of the instrument is adequate for most of the scales, although the reliability coefficient was particularly low for scores on *Empathy/Tolerance*. Consequently, this scale should be revised in the Spanish version of the ARQ, since it does not fulfil the minimum criteria for an instrument of this kind. In the original validation study of the English version, Gartland and co-workers [22] reported a much higher Cronbach’s alpha coefficient for this scale (.70), suggesting either that the construct in question needs to be redefined so as to adapt it to the Spanish context, or that the items of this scale need to be reworded. Likewise, our results suggest that the wording of items 26, 46, and 74 would need to be revised, since they have questionable discriminative capacity in this Spanish version. In light of this, it would be advisable to conduct a second study involving cognitive interviews with adolescents, with the aim of clarifying these issues related to the instrument’s validity [33]. A further point of note is that a sizeable proportion of boys and girls obtained the maximum score on the *Availability-Family* scale, thereby limiting its ability to discriminate between boys and girls with high levels of this aspect of resilience. This scale comprises just three items, two of which (items 50 and 51) are very similar, and the potential for a certain degree of overlap may lead to redundancy. Cultural factors linked to family obligations and help within kinship relations in the Spanish context may also be contributing to these results [34].

Correlations between scales from the same domain were very high in the case of *Family*, *Peers*, and *School*, indicating that the scores on one scale will generally be consistent with those on another from that domain. On the *Peers* domain, for example, the correlation between the scales *Connectedness* and *Availability* was .80. In this regard, future studies might consider whether both these scales should be retained separately as part of the ARQ or whether a single scale would be sufficient, perhaps including items from both existing scales so as to maintain representativeness, while in the process enabling a shorter version of the instrument to be produced.

Further evidence for the validity of ARQ scores comes from the relationship they showed with YSR psychopathology scores. Our results reveal an inverse relationship between resilience and psychopathological symptoms, such that the greater the resilience the fewer the symptoms, and vice-versa. Obviously, the cross-sectional nature of this study prevents us from establishing the direction of the relationship between resilience and symptoms of maladjustment, but our results do underline the presence of such a relationship and its importance in the context of intervention. Particularly noteworthy in this regard is the relationship between self-related resilience and the presence of internalizing problems. Depression and anxiety, which form the basis of this broadband syndrome scale, are closely associated with cognitive distortions and negative self-image [35], thus illustrating the relationship with the more individual aspects of resilience. A relationship between symptoms of depression and anxiety and the personal aspects of resilience has previously been demonstrated in research using the Resilience Scale for Adolescents (READ) [17]. The more moderate association we observed between resilience and externalizing symptoms was likewise reported in that study [17], since correlations between the READ and alcohol intoxication, the use of illegal drugs, theft, and violent behavior were lower than those corresponding to anxiety, depression, and suicidality. It should also be noted that the stronger relationship between resilience and psychopathology that we found in girls also has its counterpart in other studies [17].

The importance of family in relation to the development of psychopathology in adolescents has been documented previously [36], this being consistent with the fact that family connectedness appeared to play a key protective role among girls in our study. Nevertheless, our results support the importance of including multiple factors and of taking into account both personal variables and contextual resources when assessing resilience [5].

Our analysis of sex differences suggests that boys are more self-confident (*Confidence*) and less likely to experience worries and ruminations (*Negative cognition*). These differences are consistent with previous research on self-esteem [37] and rumination in adolescents [38], as well as with the fact that boys scored higher than girls on the *Personal Competence* scale of the READ [17]. These findings constitute further evidence for the validity of the scores obtained when applying the ARQ to a sample of Spanish adolescents.

### Strengths, limitations, and future research

This study is the first to adapt an instrument for assessing resilience in adolescents to the Spanish cultural context. The importance of culture in the assessment of resilience has been highlighted by other authors [25], and future studies in other countries should likewise seek to adapt standardized instruments to their own setting, thus enabling intercultural comparisons to be made. The present study does, however, have a number of limitations. The sample comprised adolescents drawn exclusively from an urban context in a specific geographical region of the country, which is relevant since research has shown that resilience varies according to the sociodemographic status of young people [14]. This aspect should be taken into account when attempting to generalize the results. A further limitation is that the process of validating the ARQ did not include any other measure of resilience that might serve as a gold standard, since it does not exist [12]. Future studies should therefore explore, among other aspects, the stability of the measurements derived from the ARQ (test-retest reliability), the instrument’s sensitivity to change in studies that involve application of an intervention, and its ability to differentiate between adolescents with high and low levels of psychosocial adaptation.

## Conclusions

In summary, the Spanish version of the ARQ is a useful tool for identifying personal characteristics associated with resilience and signs of positive engagement with family, peers, school, and the community environment. The instrument is able to measure and assess this construct with certain precision and could contribute to the development of intervention programs designed to foster these skills or resources among more vulnerable adolescents, thus promoting their future wellbeing [1].

## Additional file

Additional file 1:
**Cronbach’s alpha, item-corrected total score correlations, and factor loadings.**
