# Supplementary material for: Assessing resilience in adolescence: The Spanish adaptation of the Adolescent Resilience Questionnaire
Source: Health Qual Life Outcomes. 2015 Jul 11;13:100. doi: 10.1186/s12955-015-0259-8 (PMC4498517; doi:10.1186/s12955-015-0259-8)
Supplement: Additional file 1: — Cronbach’s alpha, item-corrected total score correlations, and factor loadings. [file 12955_2015_259_MOESM1_ESM.pdf]

Additional file 1

Cronbach's alpha, item-corrected total score correlations, and factor loadings

Domain/Scale/Item	Factor loading	Correlation item-corrected total	Domain/Scale/Item	Factor loading	Correlation item-corrected total
1. Self			Family *(continuation)*		
Confidence (α=.82)			Connectedness *(continuation)*		
1	.63	.50	48	.65	.60
6	.69	.60	49	.70	.60
7	.60	.53	Availability (α=.80)		
13	.68	.61	47	.64	.55
18	.62	.61	50	.74	.70
19	.43	.34	51	.89	.68
22	.53	.52	3. Peers		
39	.59	.58	Connectedness (α=.76)		
Emotional insight (α=.60)			52	.70	.62
4	.27	.19	54	.45	.46
10	.31	.31	57	.50	.48
14	.38	.26	61	.56	.49
16	.66	.36	63	.43	.35
26	.22	.27	65	.54	.46
27	.32	.34	66	.70	.53
36	.61	.37	Availability (α=.73)		
40	.32	.30	53	.61	.54
Negative cognition (α=.73)			55	.62	.49
2	.33	.31	56	.58	.50
5	.33	.21	58	.62	.48
8	.48	.54	59	.44	.40
11	.63	.43	60	.30	.24
20	.59	.60	62	.45	.36
30	.56	.40	64	.53	.48
32	.62	.48	4. School		
38	.48	.44	Supportive environment (α=.78)		
Social skills (α=.70)			67	.66	.63
12	.47	.25	68	.54	.58
15	.59	.43	71	.53	.54
21	.54	.55	73	.43	.37
24	.57	.52	77	.67	.42
29	.40	.29	78	.71	.53
31	.43	.42	79	.41	.38
33	.49	.33	81	.50	.47
34	.38	.27	Connectedness (α=.71)		
Empathy/Tolerance (α=.38)			69	.53	.56
3	.53	.22	70	.47	.40
9	.26	.25	72	.61	.34
17	.21	.20	74	.05	.08
23	.16	.12	75	.65	.42
25	.26	.09	76	.59	.61
28	-.09	.04	80	.53	.51
35	.20	.18	82	.37	.30
37	.27	.21	5. Community		
2. Family			Connectedness (α=.82)		

Domain/Scale/Item	Factor loading	Correlation item-corrected total	Domain/Scale/Item	Factor loading	Correlation item-corrected total
Connectedness (α=.84)			83	.70	.64
41	.76	.69	84	.59	.55
42	.63	.62	85	.55	.41
43	.78	.69	86	.74	.70
44	.70	.69	87	.68	.63
45	.71	.62	88	.72	.64
46	.22	.17			
